# Children and divorce: A rapid review targeting cognitive dissonance, in the context of narrative therapy

**DOI:** 10.1177/13591045251314908

**Published:** 2025-01-13

**Authors:** Christopher Lie Ken Jie, Joanne Jessen Yramategui, Richard Huang

**Affiliations:** The Schulich School of Medicine & Dentistry, 70384 Western University, Canada

**Keywords:** Cognitive dissonance, children, divorce, narrative therapy, family mediation, review

## Abstract

Today, for divorcing parents, the social norms of “good” parenting appear to impose obligations to “fight” for shared custody of their children. However, this may intensify conflicts experienced by their children in the form of cognitive dissonance. Authors conducted a rapid review to explore children’s experiences of divorce (ages three to 12 years old) in the context of narrative therapy, in order to uncover the mechanism of cognitive dissonance. Four databases of Scopus, PsychINFO, Family and Societies Studies Worldwide, and PubMed were searched for literature in the last 10 years. Results included 11 study articles, one policy brief, and one book chapter, representing the experiences of 1169 children from seven developed countries/regions. Our findings suggest four themes associated with cognitive dissonance, whereby the first three represent the formation of harmful perceptions of cognitive dissonance resulting from divorce. The fourth represents the children’s coping strategies to reduce their cognitive dissonance. We advocate that family mediators consider narrative therapy targeting cognitive dissonance as a means of repairing disruptions to family coherence. In this regard, we recommend that future research explore the consequences of children’s confrontation of their cognitive dissonance in narratives found to be prevalent in children’s experiences of divorce.

## Plain language summary

Children who experience parental rejection during divorce: Would targeting children’s conflicting beliefs in stories of their relationships help them to better adjust?: Divorce can be emotionally traumatic on children, especially in the early years between three and 12 years old. However, not all children that experience divorce are traumatized. Rather, children that experience parental rejection or avoidance as part of divorce, can be helped through telling their stories, to make sense and transform their experiences. We were guided by a question of whether children going through parental divorce, and whom hold two contradicting ideas (like love and hate towards one or more parents), might be helped when they talk about their conflicting perceptions with a therapist. In this paper, we explored children’s storied literature in the last 10 years to better describe children who talked about their conflicting ideas in their relationships during divorce, and what were the consequences for them. We found that loyalty conflicts were prevalent in their relationships with parental figures, but that these were not only emotionally harmful to their adjustment, but could also help them repair their relationships. In the latter case, family therapists drew on children’s contradicting beliefs to help children work through them. This strategy shifted children’s moral obligation from their parents to themselves, and helped them find alternative ways to cope. We recommend family therapists draw out conflicting perceptions in children’s storied experiences of divorce, to help them reconsider their moral obligations and forge better working alliances in their relationships.

## Introduction

Divorce or parental separation/alienation/estrangement is a common phenomenon in North America and Europe [(affecting on average approximately 42–53% of marriages) World Population Review, 2024], although it has declined slightly in the last four decades (Demir-Dagdas, 2021). While many children eventually establish a sense of normalcy after divorce, a significant percentage become entrenched in prolonged family conflict; a prevalent “caregiver-child relationship problem” shaping ones’ health (World Health Organization, 2024). As such, divorce may be associated with serious household dysfunction, abuse, and neglect stemming from the disruption of early childhood relationships to trusted parents (Fujiwara, 2022). This lays the foundations for developing attachment anxiety (i.e., fear of abandonment and lack of trust that others will respond to their needs), disorganized attachment (i.e., combination of anxiety and avoidance in relationships), post-traumatic stress disorder and/or other forms of psychopathology, such as depression (Lim et al., 2020). In addition, children (as compared to adolescents) are more vulnerable to divorce negatively impacting their socioeconomic status and generating a propensity towards substance use, such as alcohol (Demir-Dagdas, 2021).

Divorce is rarely a single event but rather is a process of disruptions that result in a cascade of effects across multiple phases, including pre-separation, separation, and post-separation/post-divorce (D’Abate, 2016). Amid the politicization and legal interplay of divorce is a growing recognition that the child’s voice ought to take “center stage,” honoring their right to be heard (Brand et al., 2017). However, in high-conflict disputes, parental interests tend to diverge, and thus, parents ought not be solely responsible to decide on what is “best” for their children (Eekelaar, 2020). As such, divorce mediation is deemed more appropriate to resolve disputes, rather than adversarial adjudication in family court (Ellis, 2022).

Divorce mediation refers to a process in which parents meet with trained persons (in psychotherapy, counseling, law, or conflict resolution) and create a written agreement about custody and visitation in the best interests of the child (Tveit et al., 2023). While there exist many models of mediation, its success depends on its capacity to create therapeutic conditions of emotional safety that facilitate children and their families to make sense, and integrate their relational experiences, into an account that helps them resolve their disputes (Lohvinenko et al., 2021). Indeed, mediation models following positive parenting behaviors may improve child adjustment post-divorce (van Dijk, et al., 2020).

### Narrative therapy for children in divorce mediation

Narrative therapists can act as a trusted mediator who will come to the child(ren)’s aid to resolve family disputes; ideally, a compassionate figure, who offers “corrective and replicative scripts” to address the child(ren)’s fears of rejection and abandonment, or fears of disappointing oneself or others (Dallos & Vetere, 2014, p. 496). Narrative therapy is based on the assumption that human life has a storied structure and the intentional efforts to make sense of this helps organize human action (Wallis et al., 2011). Indeed, children draw on early memories (beginning at age three years old), and develop autobiographical memory by age four (Middleton, 2017). This age coincides with their capacity to recall and describe past traumatic events (Middleton, 2017). By ages six to nine years old, children can convey their traumatic stories in quite accurate detail (Middleton, 2017).

Narrative therapists map out children’s stories in ways that help them “see” their problems and make sense of them within the larger context of cultural, social, historical, and political influences (Jørgensen et al., 2024). This enables children to “externalize” and deconstruct dominant storylines to reframe their perspectives (a construct called “reauthoring”) so that they may negotiate a new relationship with their problems (“unique” outcomes”), including distancing or reconstructing their situation as one residing partially, in social structures (e.g., socio-economic class, ethnicity, the legal system) (Wallis et al., 2011). However, narrative therapy reports mixed outcomes for children, in part because it is criticized as time-consuming and requires specialized education and practice (Jørgensen et al., 2024), and its active mechanism of core components are eclectic and unclear (Wallis et al., 2011). Moreover, Wallis et al.’s (2011) review of narrative therapy recommends further description integrating theory and action to move the approach forward based on “practice-based evidence.”

### The current study: Targeting cognitive dissonance

Today, for divorcing parents, the social norms of “good” parenting appear to impose obligations to “fight” for shared custody of their children (Bertelsen, 2023). However, this may intensify identity conflicts experienced by children in the form of cognitive dissonance, defined as instances when one’s attitudes, beliefs, and/or values contradict (are dissonance) with their behaviors (Cooper, 2019). In the process of divorce, we suggest that cognitive dissonance may occur when children feel pressured to take sides between estranged parents and act against their beliefs, a construct called “triangulation; ” alternatively, children may feel dissonance when they experience “role diffusion; ” that is, they experience discomfort when put in the role of parenting, either for practical or emotional support (van Dijk et al., 2020).

The authors suggest that experiences of cognitive dissonance reflects the variation in interparental conflict post-divorce (van Dijk et al., van der Valk, Deković, et al., 2022). Moreover, cognitive dissonance may be resolved if a child learns by their parents/ caregivers s/he is not responsible for the that interparental conflict (Cooper, 2019). Thus, authors posit that the ways children cope with cognitive dissonance is key mechanism in the development of psychopathology from parental divorce experiences.

To better understand the mechanism of cognitive dissonance, authors conducted a rapid review to explore children’s experiences of divorce in the context of narrative therapy; specifically, by drawing on literature that utilized a narrative therapy approach to inform therapeutic practice. We focused on children aged three to 12 years as our target population, based on research that they experience significant problems in their developmental milestones and identity transitions while adapting to the societal norms associated with divorce (Pires & Martins, 2021). Moreover, children’s awareness of, and response to, custody threats appear to be heightened in early childhood (Pires & Martins, 2021).

## Method

We conducted a rapid literature review guided by the Cochrane Rapid Reviews Methods Group (Garritty et al., 2021). The research question was: *Can targeting cognitive dissonance, in the context of narrative therapy, help children better cope with divorce?* The rapid review study design was selected for its potential to synthesize current evidence in “an accelerated process” (i.e., four months) and to “produce evidence for stakeholders in a resource-efficient manner” (Garritty et al., 2021, p. 15). Having identified narrative research and literature pertaining to children’s experiences of divorce, we searched for the inclusion of cognitive dissonance or one of its associated elements* (details in search strategy).

Given our narrow/niche research focus and its emergent nature in literature, we considered gray literature to be a valuable resource that could enhance our opportunities to find relevant studies and to provide a comprehensive view of all available evidence (Mahood et al., 2014). In addition, given the short timeline that we had to complete the rapid review, we did not submit a protocol to Cochrane or to PROSPERO for registration.

### Search strategy

The keywords used in the search strategy were selected based on the PICO framework representing population, intervention, context, and outcome. The systematic search was limited to four main databases: Scopus, PsychINFO, Family and Societies Studies Worldwide, and PubMed. The search combined keywords, MeSH terms, and synonyms with Boolean operators (AND/OR) in all fields. Specifically, the search strategy combined (“childhood”) AND (“narrative OR storytelling OR story OR externalization OR internalization”) AND (“divorce”) AND (*“dissonance OR acceptance OR trivialization OR compliance OR conflict OR justification OR avoidance OR denial OR adjustment”). [(*These terms refer to elements of cognitive dissonance, as conceptualized by a literature review published by Yahya & Sukmayadi, 2020)].

### Study selection

**Figure 1.**
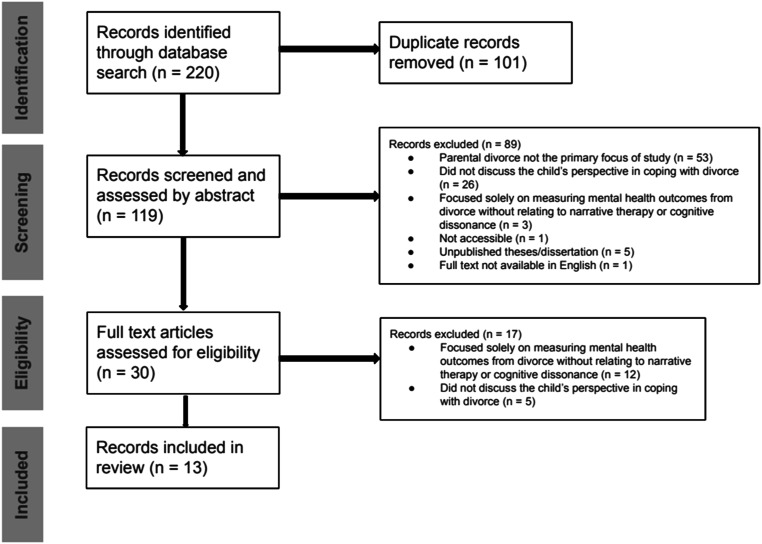
PRISMA flow diagram showing selection process used for 2024 rapid review of literature of divorce, narrative therapy and cognitive dissonance (template adapted from https://www.prisma-statement.org/PRISMAStatement/FlowDiagram?AspxAutoDetectCookieSupport=1).

Two researchers (CLKJ, JJY) conducted a pilot exercise to independently screen titles and abstracts according to *inclusion criteria:* (i) a focus on children that experienced parental divorce between the ages of three and 12 years; (ii) a narrative discourse or association with storytelling, or narrative strategies of internalization and externalization of negative attitudes and behaviors; (iii) involving an element of cognitive dissonance* and/or behaviors exhibited from the child in the context of coping with divorce to reduce internal conflict; and (iv) published in the last 10 years. *Exclusion criteria were:* (i) focused solely on measuring mental health outcomes from divorce without relating to narrative therapy or cognitive dissonance; (ii) not available in English; (iii) unpublished theses/dissertations; and (iv) did not discuss the child’s perspective in coping with divorce. A third researcher (RH) assisted in resolving any selection discrepancies that arose between the two researchers (CLKJ, JJY). All selected abstracts were then subject to full-text screening by two researchers (CLKJ, JJY) to confirm inclusion. The web tool Rayyan was used for data management and organization. The PRISMA template was used to report the results of our screening (Figure 1).

### Data extraction and synthesis

One reviewer (CLKJ) performed descriptive extraction of the data of all included articles, while a second reviewer (JJY) checked for the correctness and completeness of the extracted data located in a table with the following information: article title, author names, publication year, country, sample, study design, narrative component, and cognitive dissonance component. Except for grey literature, included studies were assessed independently by 2 researchers (CLKJ, JJY) through quality appraisal using the following study design appraisal tools: Critical Appraisal Skills Programme (for cohort and qualitative studies), Mixed Method Appraisal Tool (for mixed methods studies), and the Critical Appraisal Tool (for cross sectional studies) (See Table 1). All data were synthesized thematically to conceptualize and narrate the data. While a single reviewer (CLKJ) interpreted all evidence, a second reviewer (JJY) verified all judgments.

**Table 1.** Brief description of the included studies.

Author(s), year	Country	Sample	Design	Narrative component	Cognitive dissonance component	Quality appraisal
Artioli et al. (2014)	New Zealand	200 young adults	Mixed methods	Memory coherence	Early autobiographical memories	Satisfactory
Bacro and Macario de Medeiros (2021)	France	64 preschoolers (ages 3–6)	Cross-sectional	Attachment story completion task	Developing coping strategies	Good
Bezuidenhout et al. (2018)	South Africa	5 first graders	Qualitative	Narrative approach	Resilience, agency, and meaning-making	Good
Blow and Daniel (2016)	United Kingdom	N/A	Commentary	Narrative approach	Constructing their own narrative	N/A
Brand et al. (2017)	South Africa	5 children (ages 9–10)	Qualitative	Narrative approach	Informing therapeutic approaches	Good
Christopher et al. (2017)	United States	240 young adults	Cohort	Narrative approach	Didactics, modeling by group leaders and role-playing	Good
D’Abate (2016)	North America	N/A	Policy brief	Family narrative perspective	Creating desirable family stories	N/A
O’Hara et al. (2019)	United States	141 children (ages 9–18)	Randomized controlled trial	Self-measures of parenting time/quality and interparental conflict	Explored optimal parenting time	Good
Pantelis et al. (2015)	Greece	2 young adults (ages 21, 24)	Qualitative	Narrative approach	Divorce “attacked” ability to change	Good
Rejaän et al., (2022)	The Netherlands	251 adolescents	Cross-sectional	Narrative approach	Being caught between parents	Satisfactory
Stokkebekk et al. (2019)	Norway	9 adolescents	Qualitative	Narrative approach	Children negotiate roles during parental conflict	Excellent
Van Dijk et al., (2020)	The Netherlands	135 children (mean age 12)	Cohort	Exploration of self-blame	Self-blame associated with loyalty conflicts	Excellent
van Dijk, van der Valk, Deković, and Branje (2022)	The Netherlands	117 children (mean age 12)	Cohort	Sibling relationships	Sibling relationships are complementary to self-esteem	Excellent

Quality appraisals were conducted using Critical Appraisal Skills Programme, Mixed Methods Appraisal Tool, and the Critical Appraisal Tool. Scores were given as 1 for meeting criteria, 0 for partial, and −1 for not meeting. These were converted to percentages (−100%–100%). Judgements were based on research clarity and methodology. Eleven studies were appraised, excluding grey literature. Ratings: 0% or below = unsatisfactory, 10%–50% = satisfactory, 51%–80% = good, 81%–100% = excellent.

## Results

Thirteen items of literature were included: 11 study articles, one policy brief (D’Abate, 2016), and one book chapter (Blow & Daniel, 2016). The literature originated from seven countries/regions, representing some degree of diversity: two from the United States (Christopher et al., 2017; O’Hara et al., 2019), three from the Netherlands (Rejaän et al., 2022; van Dijk, van der Valk, Buist, et al., 2022; van Dijk, van der Valk, Deković, & Branje, 2022), two from South Africa (Bezuidenhout & Theron & Fritz, 2018; Brand et al., 2017), one from New Zealand (Artioli & Reese, 2014), one from Greece (Pantelis et al., 2015), one from France (Bacro & Macario de Medeiros, 2021), and one from Norway (Stokkebekk, Iversen & Hollekim et al., 2019). The policy brief was written for the North American context (D’Abate, 2016). Cultural diversity was limited to descriptive accounts of participants’ contexts in each study, following its research design.

Taken together, the literature represented 1169 children’s experiences of divorce; most participants recounted their experiences in early adolescence [(*n* = 1095) (Artioli & Reese, 2014; Christopher et al., 2017; O’Hara et al., 2019; Pantelis et al., 2015; Rejaän et al., 2022; Stokkebekk, Iversen & Hollekim et al., 2019; van Dijk, van der Valk, Buist, et al., 2022; van Dijk, van der Valk, Deković, & Branje, 2022). The book chapter by Blow and Daniel (2016) did not consistently specify the children’s ages.

The included studies represented five study designs: cohort [(*n* = 3) (Christopher et al., 2017; van Dijk, van der Valk, Buist, et al., 2022; van Dijk, van der Valk, Deković, & Branje, 2022)], cross-sectional [(*n* = 2) Bacro, Macario de Medeiros, 2021; Rejaän et al., 2022], qualitative [(*n* = 4) (Bezuidenhout & Theron & Fritz, 2018; Brand et al., 2017; Pantelis et al., 2015; Stokkebekk, Iversen, Hollekim, et al., 2019)], mixed method [(*n* = 1) (Artioli & Reese, 2014)], and a randomized controlled trial [(*n* = 1) (O’Hara et al., 2019)]. The book chapter by Blow and Daniel (2016) included narrative descriptions of children who had experienced divorce during childhood. After quality appraisal, 2 studies were rated satisfactory, 6 studies were rated good and 3 were rated excellent. See Table 1 for a description of each study.

### Thematic results

Our analysis identified four themes associated with cognitive dissonance. The first three themes represent the formation of harmful perceptions among children of cognitive dissonance from divorce and the fourth theme represents the adjustment process by which the children coped and mitigated their cognitive dissonance. In that which follows, we shall explore each theme in detail.

#### Divorce does not need to be a threat

Divorce is associated with strong preconceived negative ideas with respect to its effects on children as a violation of their family system (van Dijk, van der Valk, Deković, & Branje, 2022), which is perpetuated by social norms (Bacro, Macario de Medeiros, 2021; Brand et al., 2017; D’Abate, 2016; Rejaän et al., 2022), and situates children as victims of divorce (Brand et al., 2017; D’Abate, 2016; Pantelis et al., 2015; Rejaän et al., 2022; van Dijk, van der Valk, Deković, & Branje, 2022; Blow & Daniel, 2016; Stokkebekk, Iversen, Hollekim, et al., 2019). Indeed, judges use the child’s voice when determining custody and visitation rights (Blow & Daniel, 2016; van Dijk, van der Valk, Buist, et al., 2022). Consequently, with limited psychosocial services, children’s voices becomes part of a greater legal agenda to reduce the burden of high interparental conflict on the legal system (Blow & Daniel, 2016). Paradoxically, as the child’s voice takes more narrative prominence in parental legal battles, the children can become more silent (Bezuidenhout et al., 2018; Blow & Daniel, 2016; Brand et al., 2017; Pantelis et al., 2015). Therefore, social norms and legal battles can frame divorce as a threat to children.

#### Divorce perceptions depend on the child’s life stage

According to Artioli et al. (2014), the narrative coherence of divorce events, defined as recall of “the when, where and what” of the divorce event and its significance to the narrator, was shown to be unexpectedly better when the divorce occurred earlier in a child’s life (as young as three years old), due to less exposure to parental conflict. Indeed, research findings support the notion that children’s distress may be associated with a lack of narrative coherence in conflicting perceptions of their parents in middle to older childhood (Bacro & Macario de Medeiros, 2021; Blow & Daniel, 2016).

#### Loyalty conflicts

Loyalty conflicts from triangulation occur from the active involvement of the child within negotiations of parental entitlement (Blow & Daniel, 2016; Rejaän et al., 2022; Stokkebekk, Iversen, Hollekim, et al., 2019; van Dijk, van der Valk, Deković, & Branje, 2022), inducing cognitive dissonance for children. This theme encompasses two subthemes: (i) obligation to choose a side, and (ii) dissociation from parental divorce, which reflects a manifestation of children’s cognitive dissonance.

#### Obligation to choose a side

Children commonly found themselves caught in the middle of conflicting polarized storylines (Blow & Daniel, 2016; Stokkebekk, Iversen & Hollekim, 2019) that largely took the form of a parent wanting to “fight for parent equality,” or “fighting for the child’s rights of choice,” which involved labeling the other parent as an oppressor. This positioned the child as an independent agent and the parents as “neutral and supportive recipients” (Stokkebekk, Iversen, Hollekim, et al., 2019). Children reported feeling like an “object,” “messenger,” “ally” or “pawn” in parental conflict or “solely responsible” for themselves (Rejaän et al., 2022; Stokkebekk, Iversen, Hollekim, et al., 2019; van Dijk, van der Valk, Deković, & Branje, 2022). Consequently, children found themselves unable to move beyond hostility and exposure to negative information about the other parent (D’Abate, 2016; Stokkebekk, Iversen, Hollekim, et al., 2019) generating cognitive dissonance.

#### Dissociating from parental divorce

Higher levels of empathy in children appeared to induce greater levels of cognitive dissonance in them, as they understood both parents’ emotions (van Dijk, van der Valk, Deković, & Branje, 2022). These storylines were often characterized by bitterness and resentment, particularly when parents were unable to take responsibility for their actions (Pantelis et al., 2015; Stokkebekk, Iversen, Hollekim, et al., 2019). Consequently, children expressed emotional exhaustion from prolonged conflict (Brand et al., 2017; Pantelis et al., 2015; Stokkebekk, Iversen, Hollekim, et al., 2019). Later, children expressed grief when they eventually felt forced to remove themselves from one parent in the interest of protecting themselves (Pantelis et al., 2015; Stokkebekk, Iversen, Hollekim, et al., 2019).

#### Agency over coping with cognitive dissonance

This theme describes children’s need to negotiate accountability with others in the interest of reauthoring their own new narrative. This involved constructing new alliances to support a shift in parents’ sense of moral obligation toward their children’s own well-being. Despite parental conflict, children generally did not wish to adhere to narratives of blame directed toward their parents (Blow & Daniel, 2016). When children removed themselves from an obligation to repair their family dynamics, they reported developing feelings of self-blame, guilt, sadness, and jealousy (Christopher et al., 2017; Stokkebekk, Iversen, Hollekim, et al., 2019). Hence, commitment to a desirable, enduring family story required that working alliances be forged between parents (D’Abate, 2016; Pantelis et al., 2015) to reduce the children’s cognitive dissonance and allow them to dissociate themselves from parental divorce (D’Abate, 2016; Stokkebekk, Iversen, Hollekim, et al., 2019).

Therapists, extended family, or step-parents were found to be helpful in supporting such working alliances by playing the role of a “stabilizing third” and mobilizing family resources (e.g., siblings, friends, and neighbors) to deconstruct narratives that frame children as “victims” (Bezuidenhout & Theron & Fritz, 2018; Brand et al., 2017; Pantelis et al., 2015; Rejaän et al., 2022; Blow & Daniel, 2016; van Dijk, van der Valk, Buist, et al., 2022; Stokkebekk, Iversen, Hollekim, et al., 2019). Moreover, this allowed children to shift their moral obligation to one of self-agency, based on family interdependence, both inside and outside their family lives (Brand et al., 2017; O’Hara et al., 2019; Stokkebekk, Iversen, Hollekim, et al., 2019).

## Discussion

As in past literature about divorce, when adult children remove themselves from both biological parents after divorce, this can lead to negative consequences on their wellbeing, described as negative consonance by Hornstra et al. (2022). Indeed, negative consonance can occur during childhood and persist into adulthood known as “chronic dissonance,” associated with depression and loneliness (Hornstra et al., 2022, p. 500). In other words, how children learn to separate themselves from feeling responsible for their parents’ divorce and conflict is key to coping with their cognitive dissonance as part of the divorce process (Priolo et al., 2019). Our results revealed if children could negotiate their accountability for their parents’ divorce with a stabilizing third, separate from parental triangulation and loyalty conflicts, they could construct a “new,” more coherent narrative.

Like past literature on cognitive dissonance, our results support its potentially detrimental effects as well as its potential benefits for children (Hornstra et al., 2022). Narratives of divorce reveal how a child resolves their cognitive dissonance can lead to both positive and negative attitudes towards themselves and to their parents. On the one side, our findings indicate that in order to resolve their cognitive dissonance from loyalty conflicts, children may blame themselves as a means of making sense of divorce events—in other words, they may conform to the belief that “people get what they deserve,” known as the “just world belief” (Aguilar et al., 2022, p. 17). According to Aguilar et al. (2022), people are motivated to blame victims for their misfortune to preserve their own perception of a “just world.” Such that, when something bad happens to an innocent victim, this violates this belief and generates cognitive dissonance, leading to denial of victimization, minimization of victimization and victim blaming as forms of “cognitive restoration” (Aguilar et al., 2022, p. 5). Similarly for children involved in triangulation from divorce, they may show similar processes of cognitive restoration, including blaming themselves for the divorce. From our results, this may reflect an attempt to explain their active involvement in loyalty conflicts while not adhering to blame narratives towards their parents (Blow & Daniel, 2016). Further, they may cope in ways that dissociate themselves from the divorce and inadvertently generate internalized stigma, denial, minimization of their feelings, and/or a tendency to avoid confrontation of interpersonal conflict (Pantelis et al., 2015). Indeed, avoidance of cognitive dissonance, such as avoiding a parent that creates loyalty conflicts, may be a protective measure, yet it may exert a detrimental impact on their relationships (Aguilar et al., 2022).

On the other side, the results of our review indicated that divorce need not be a threat, as it is socially constructed by social norms and legal disputes. Aligned with perceptions that divorce may be a normal part of life (Fučík, 2020), our results support narrative therapy can facilitate coping with cognitive dissonance. Our results suggest that mediators could act as “stabilizing” third persons to pursue working alliances between parents, such that they could take responsibility for their actions and resolve their disputes. Hence, the threat of divorce could be mitigated for children, and they may have agency to reauthor their stories. Indeed, we suggest that mediators following narrative therapy might afford children space to identify moral obligations to themselves, separate from their parents. In doing, so, children might transform their beliefs from former one that they were “victims,” to empowered persons with resources (competencies) as a result of their divorce experience.

### Future directions

We suggest research exploring the mechanism of cognitive dissonance has the utility to inform “evidence-of-narrative practice,” and thereby may expand its utility for family mediators of divorce. According to Rafti (2019), narrative therapy fits well with the therapists’ arsenal of tools, including, among other approaches, the structured-negotiation model, transformative model, and facilitative mediation. We recommend the use of narrative therapy may depend upon, if and how, cognitive dissonance occurs (i.e., in triangulation and role diffusion) and the degree it does in post-divorce parenting (high/low conflict) and the parent-child relationship. Hence, we argue that a focus on revealing children’s patterns of cognitive dissonance may enhance the efficacy of narrative therapy; to help harness the drive to resolve the discrepancies and effect outcomes, not as a single event, but as a process of socialization that shapes children’s life trajectory.

Our study findings also point to micropolitics at play in the court system. Its legal and administrative culture may play a role in how children are socialized to experience divorce as a threatening process. Eekelaar (2020) asserts that the key may be the early establishment of parental common interests in achieving what is “best” for their children. As such, lawyers may benefit from mediation training in narrative therapy, which situates them in an advisory role to facilitate the child’s interests and gives them additional means of achieving it (Eekelaar, 2020). To do this, lawyers may work with family mediators to consistently enforce a stabilizing alliance that resists situating children as “victims,” but rather helps them to negotiate their agency to resolve their cognitive dissonance, interdependent with their parents as they undergo divorce.

### Strengths and limitations

Our study’s main strength lay in its ability to identify a potentially universal cognitive schema describing the adjustment process post-divorce in children. Targeting cognitive dissonance within narrative therapy may be a key mechanism to prevent traumatic experiences such as divorce from causing adverse health outcomes.

Findings related to cultural diversity were not predominant in our review. Family mediators must take children’s cultural diversity into account to ensure therapy is delivered in culturally appropriate ways (e.g., drawings) (Rafti, 2019). With that said, while the sample represented some degree of cultural diversity (i.e., seven countries) it also constitutes a limitation. A study by Smith-Greenaway and Clark (2017) found children of divorced mothers in African countries experienced greater morbidity and mortality where divorce is less reported, and thus less accepted. Researchers found some evidence to indicate that better outcomes for children were due to the greater social acceptance of marital divorce in some regions, rather than the mothers’ education and wealth (Smith-Greenaway & Clark, 2017). Given that social norms serve as a moderator of one’s cognitive dissonance, our study findings may support conjectures that one’s social environment may be a key driver for cross-contextual variations in the generation of childhood disadvantage associated with divorce (as posited by Smith-Greenaway & Clark, 2017). However, the social environment and norms of one’s culture was not the focus of our study, and as such, our findings may not be generalizable to differing social contexts of children in underdeveloped countries, nor are our findings generalizable to children aged over 12 years.

## Conclusions

In sum, our review is among the first to reveal a connection between cognitive dissonance and children’s experience-based narratives of divorce. Specifically, our findings suggest that the deconstruction of children’s conflicted loyalty offered them the opportunity to reflect on their self-blame, guilt, and sadness. In doing so, they were confronted by tensions of when they felt obliged to be a responsible “ally” to one parent while also being treated as their “pawn.” Given support to reframe their moral obligation to themselves, they were enabled to renegotiate the quality of their relationships with their families. Indeed, our results support Aguilar et al.’s (2022) conclusion that manifestations aimed at revealing children’s cognitive dissonance may “inoculate them” against future relational stressors.

Aguilar et al. (2022) describes a process whereby the confrontation of cognitive dissonance—manifested as ones’ beliefs contradicting their behaviors—can offer individual skills to reappraise and resolve their tension. In doing so, it may imbue them with the capacity to resist perceptions of them as “victims” in future similar scenarios. Moreover, we advocate that family mediators consider narrative therapy targeting cognitive dissonance as a means of remedying disruptions to family coherence. In this regard, we recommend that future research explore the effects of children’s confrontation of their cognitive dissonance in narratives found to be prevalent in children’s experiences of divorce.
